# Ligand effect on controlling the synthesis of branched gold nanomaterials against fusarium wilt diseases†

**DOI:** 10.1039/d2ra05478g

**Published:** 2022-11-07

**Authors:** Francis J. Osonga, Gaddi B. Eshun, Omowunmi A. Sadik

**Affiliations:** BioSensor Materials for Advanced Research and Technology (The BioSMART Center), Chemistry and Environmental Science Department, New Jersey Institute of Technology, University Heights 161 Warren Street Newark NJ 07102 USA sadik@njit.edu

## Abstract

The widespread wilt disease caused by *Fusarium solani* spp is a pressing problem affecting crop production and intensive farming. Strategic biocontrol of *Fusarium solani* spp using phytochemical mediated nano-materials is eco-friendly compared to harsh synthetic fungicides. The present study demonstrates the comparative dose effects of QPABA-derived branched gold nanomaterial (AuNF) and quercetin-mediated spherical gold nanoparticles (s-AuNPs) against *Fusarium solani* spp. Quercetin-*para* aminobenzoic acid (QPABA) was synthesized using reductive amination by reacting *para*-aminobenzoic acid with quercetin in an eco-friendly solvent at 25 °C. The structure elucidation was confirmed using ^1^H and ^13^C-NMR. TLC analysis showed that QPABA (*R*_f_ = 0.628) was more polar in water than quercetin (*R*_f_ = 0.714). The as-synthesized QPABA serves as a reducing and capping agent for the synthesis of gold nanoflowers (AuNFs) and gold nanostars (AuNSs). The UV-vis, XRD, and TEM confirmed the SPR peak of gold (550 nm) and gold element with a particle size distribution of 20–80 nm for the nanostars respectively. AuNFs exhibited a significant (*P* < 0.05) inhibitory effect against *F. solani* in a dose-dependent manner using Agar well diffusion. Nevertheless, spherical-AuNPs were not effective against *F. solani*. The inhibitory effect was influenced by the size, dose treatment, and particle shape. The minimum inhibitory concentration (MIC) value of AuNFs was 125.7 ± 0.22 μg mL^−1^. Our results indicate that AuNFs show considerable antifungal activity against *F. solani* as compared to spherical AuNPs. This study shows a greener synthesis of gold anisotropic nanostructures using QPAB, which holds promise for the treatment of fungal pathogens impacting agricultural productivity.

## Abstract 2

The widespread wilt disease caused by *Fusarium solani* spp is a pressing problem affecting crop production and intensive farming.

## Graphical abstract

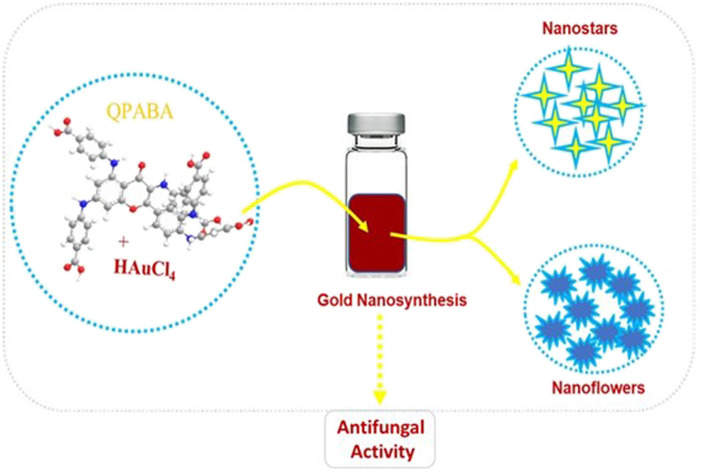

## 1 Introduction

Crop production affected by soil-borne diseases is a major agricultural problem worldwide*. F. solani* produces mycotoxins that endanger plants and animals^1–3^ and causes wilt disease in perennial crops such as guava and tomatoes with an apparent low production and economic loss. To control the soil-borne fungi (*F. solani*) in perennial crops, synthetic chemicals such as thiophanate methyl^2^ are effective treatments against *Fusarium solani*.^1,3^ However, synthetic chemicals are hazardous and impact soil fertility. In this regard, plant-derived nano-materials are a greener and more cost-effective treatment against fungi diseases. This study aims to compare the application of water-soluble QPABA-derived gold nanoflowers and s-AuNPs for the treatment of *F. solani*.

Flavonoids act as therapeutic agents against diseases such as cancer, and metabolic disorders^4–6^ quercetin is a polyphenol with an antioxidant property but low water solubility.^7,8^ The need to improve the water solubility of quercetin is important for its applications.^9,10^ Polar amino acid *para*-aminobenzoic acid (PABA) attached to quercetin improves water solubility and enhances its physiochemical properties. PABA is a naturally occurring amino acid with promising applications in nanoparticle synthesis.^11–16^ Hence the idea is to synthesize QPABA to form a highly water-soluble reducing and capping agent for greener anisotropic nanostructures at room temperature.

Up until now, advanced methods for the synthesis of gold nanoparticles include biological, chemical, and physical methods such as lithography. These methods violate the principle of green chemistry by using excessive heat, and organic solvents.^17^ Plant-based synthesis of gold nanoparticles is proven to be safe and eco-friendly, with an ability to control the morphology and sizes of the resultant nanoparticles based on controlling the concentration of the reactants. This work promotes the use of modified quercetin as a natural shape-directing agent for the synthesis of gold nanoflowers and gold nanostars without any heat and toxic surfactant.

Nanomaterials have played a significant role as antifungal agents owing to their unique size and shape-dependent properties.^3,18,19^ The management of fungal diseases using different nanomaterials (copper, silver, and zinc oxide nanoparticles) is majorly considered cost-effective and eco-friendly, especially when synthesized by green methods.^3,18^ Although antifungal nanoparticles such as silver nanoparticles are undoubtedly effective against several fungal diseases, they are harsh and toxic. Among nanomaterials, gold is selected due to its biocompatibility and no toxicity.^19^

Flower-like nanostructures with sharp edges have multiple applications due to their high localization surface plasmon modes.^20,21^ Some factors that control the size and shape include the stabilization effect of the capping agent/solvent pair, the intrinsic reactivity of the metal precursor, and reaction temperature. Some methods for synthesizing gold nanostars and nanoflower production include seed-mediated and one-pot methods.^22,23^ The formation of AuNFs was dependent on varying concentrations of reducing/capping agents and the gold precursor.^11,23–25^ Thus, the goal of functionalizing quercetin with *para*-aminobenzoic acid was to increase its polar solubility as a vital parameter for the aqueous synthesis of AuNFs and AuNS.

In this work, quercetin was functionalized with *para*-aminobenzoic acid *via* reductive amination to produce a new compound known as quercetin-*para*-aminobenzoic acid (QPABA). The QPABA was applied as a reducing and capping agent for the synthesis of AuNFs and AuNS using the chemical reduction technique.^26,27^ Characterization was achieved using TEM, UV-vis spectroscopy, and FTIR. We investigated the antifungal of gold NFs and spherical AuNPs against *F. solani*, using agar well diffusion assay. Our results indicate that the AuNFs showed a higher zone of inhibition against *F. solani* compared to the treatment with spherical AuNPs. The flower-like shape played an influential role in the inhibitory effects against *Fusarium solani*. This study promotes QPABA as a cost-effective and safe shape-directing agent for synthesizing different anisotropic nano-materials for the treatment of pathogenic fungi.

## 2 Materials and method

### 2.1 Reagents

Hydrogen tetrachloroaurate (HauCl_4_·3H_2_O) and *para*-aminobenzoic acid were purchased from Sigma-Aldrich, Milwaukee, WI. Methanol, sodium hydroxide (NaOH) pellets, and hydrochloric acid were purchased from Fisher Scientific, Pittsburg, PA. Anhydrous quercetin was purchased from MP Biomedicals, LLC, Solon, Ohio. Synthesis was carried out using ultrapure water (with a resistance of more than 18.2 MΩ cm). The reagents used are analytical grade with the highest purity of >97% without further purification. *Fusarium solani* M.S 52628 was purchased from the American Type Culture Collection (ATCC) (Manassas, VA). 100 mm Corning Agar plates were purchased from Sigma-Aldrich (St. Louis, MO). Potato dextrose agar (PDA) and dextrose glucose broth (DTB) were purchased from Oxoid Microbiology products (Hampshire, UK).

### 2.2 Synthesis of quercetin-*para* aminobenzoic acid (QPABA)

**Scheme 1:**
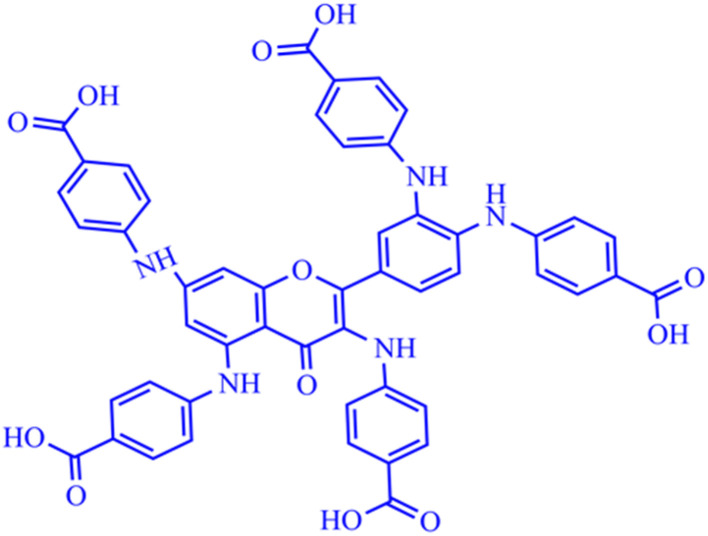
Schematic diagram of QPABA product and its IUPAC name generated from Chemdraw.

Quercetin-*para* aminobenzoic acid (QPABA) was synthesized using *para*-aminobenzoic acid and quercetin *via* a reductive amination technique. 1 mmol of QCR (300.23 mg) was weighed and dissolved in 200 ml of methanol in a 500 ml Erlenmeyer flask. Briefly, 5 mmol (137.14 mg) of *para*-aminobenzoic acid was dissolved in a 70% acetic acid and 30% water mixture. Thereafter, the *para*-aminobenzoic acid mixture was then added to the QCR mixture. 2 ml HCl was added to catalyze the reaction at the beginning of the reaction, and 8 pellets of NaOH were added to neutralize any excess HCl acid after the initial reaction. The reaction proceeded for 2 hours and was monitored by thin-layer chromatography (TLC), and then 300 mg of reducing agent (borane dimethylamine) was added, and the reaction then proceeded for 24 hours. The solvents from the reaction were extracted using a rotor vapor system. The product obtained was then purified using flash chromatography to obtain yellow solid identified as QPABA with IUPAC name (4,4′,4′′-((2-(3,4-bis((4-carboxyphenyl)amino)phenyl)-4-oxo-4*H*-chromene-3,5,7-triyl)tris(azanediyl))tribenzoic acid) as shown in Scheme 1.

### 2.3 Synthesis of AuNFs and AuNS using QPABA

One-pot synthesis was used to achieve the reduction of Au^3+^ to Au^0^ in the presence of QPABA, which served as a reducing and stabilizing agent. Before the synthesis, 1 mM of aureate solution (HauCl_4_·3H_2_O) was freshly prepared in a cleaned 50 ml vial. After that, 5 mg of QPABA was dissolved in 5 mL of ultrapure water in a cleaned separate 50 ml vial. The reaction of the AuNFs and AuNS proceeded in a clean, dry 50 ml vial using 5 mg of QPABA, 2 ml aliquots of HauCl_4_·3H_2_O, and 5 ml of distilled water. Both varying time experiments and -dependent experiments were carried out at room temperature (25 °C), accompanied by significant color changes. The spectrophotometric measurements of the samples were performed using an Agilent Technologies 8453 UV-vis spectrophotometer. AuNFs and AuNS were separated and purified *via* centrifugation at 3500 rpm for 10 minutes, and the pellets, in addition to unreacted QPABA, were removed with 18 MΩ cm resistivity ultrapure water. The same method was used to synthesize spherical AuNPs using quercetin.

### 2.4 NMR and TEM characterization

The structure of quercetin – *para*-aminobenzoic acid (QPABA) was determined using both ^1^H and ^13^C NMR Bruker 600 MHz operated through Topspin 3.0 NMR software. UV-vis spectra of the resulting QPABA-based nanoparticle solutions were determined using Hewlett-Packard 8453 UV-vis spectrophotometer from 200 to 1000 nm. The samples for TEM analysis were prepped *via* drop-casting the samples of liquid aliquots on SiO_2_-coated 300 mesh copper grids. The samples are dried thereafter. The nanoflowers' size and morphology were examined and analyzed using Axion Vision software version 4.8.2. TEM. A drop of the sample was added to the TEM grid and dried at room temperature for TEM imaging. The experiment was performed, and the images were recorded. The TEM images and size distribution measurements of AuNFs and AuNS were prepared. The XRD measurements for the samples were analyzed using a Bruker D8 Discover XRD system operating at 40 kV and 40 mA. The XRD samples were dissolved in ethanol and then mounted on sample holders for measurements. Furthermore, XRD patterns were obtained using an X-ray diffractometer (Bruker D8 Advance 800234-X-ray (9729), Germany) using Cu-Kα radiation (l ¼ 1.5178 A, 40 kV, and 40 mA). The 2-theta angle scanning range was from 5 to 80 with a scanning speed of 0.1 per second.

### 2.5 Thin-layer chromatography (TLC) analysis

Standard stock solutions of QPABA and quercetin (1 mg mL^−1^) were separately diluted in methanol to obtain 0.10 mg mL^−1^ working solutions. The working standard solutions were then spotted on the TLC silica gel 60 F254 as shown in Fig. S3.† The experiment was performed in triplicate. The plate was developed using the solvent system methanol : ethyl acetate (1 : 2 v/v) in a transparent chamber, then allowed to saturate for 15 min after reaching 7 cm. The optimal solvent for QPABA was determined by varying solvent ratios. The plate was dried and visualized under ultraviolet light (254 nm). After development, the plate was removed and dried and spots were visualized under UV light at 254 nm. The retardation factor (*R*_f_) was calculated using the formula *R*_f_ = *A*/*B*. where *A* represents the distance traveled by the solute and *B* represents the distance traveled by the solvent.

### 2.6 Fourier-transformation infrared spectroscopy (FT-IR)

The Fourier-Transform Infrared Spectroscopic (FT-IR) analysis was conducted to confirm the presence of functional groups in the structure of the synthesized QPABA. The FT-IR spectra of both quercetin and QPABA were recorded using the Shimadzu IRTracer-100 Fourier Transform Infrared Spectrophotometer (Shimadzu Corporation, Kyoto, Japan) equipped pyroelectric DLATGS detector and advanced dynamic alignment. The sample compartment was cleaned with deionized water and purged with dry air to minimize the interference with the absorbance of atmospheric water vapor. The spectrum of air recorded as a background signal was subtracted automatically using an in-built software of the instrument. The dried QPABA sample was crushed into fine particles and loaded unto the sample holder. The FT-IR spectral scans were recorded from 4000–400 cm^−1^ at a resolution of 4 cm^−1^ and 45 scans were recorded during the analysis. The dried QPABA samples were analyzed without further sample modification.

### 2.7 Agar well diffusion assay

The antifungal activity of AuNFs and AuNPs was determined by agar well diffusion method.^19^*F. solani* spores were grown in potato dextrose broth (PDA) media for 7 days. Furthermore, *F. solani* of 0.02 ml were inoculated in 20 ml of molten PDA. The culture tubes were homogenized and poured into a 100 mm corning petri dish. The culture plates solidified in a biosafety hood and then two 5 mm wells were created on the agar plate using a Cork borer. Each agar plate was loaded with AuNFs (0.10 mg mL^−1^, 0.15 mg mL^−1^, and 0.20 mg mL^−1^) in one experimental setup. In a second setup, each plate with wells was treated with spherical AuNPs (0.10 mg mL^−1^, 0.15 mg mL^−1^, and 0.20 mg mL^−1^) Quercetin dissolved in water was used as a positive control. The efficacy of AuNFs and spherical AuNPs nanoparticles against the *F. solani* were determined in comparison to the control. The plates were incubated at 25 ± 2 °C for 5 days. The antifungal activity was estimated using measuring the zone of inhibition. The mycelial growth was measured 5 days after incubation. The inhibition percentage was calculated usingAntifungal inhibition (%) = [(*S*_b_ − *S*_a_)/*S*_b_] × 100where *S*_a_ is the diameter of the growth zone around the wells (mm) and *S*_b_ is the diameter of the growth zone in the control well (mm).

### 2.8 Determination of minimum inhibitory concentration (MIC)

Broth dilution assay was adapted to determine the minimum inhibitory concentration as reported elsewhere. It is the lowest concentration of the AuNFs/AuNPs to completely inhibit any growth. The antifungal effectiveness was determined against 10^5^ colony-forming units mL^−1^.

### 2.9 Statistical analysis

Statistical results were reported as mean ± standard deviation (SD). Experimental results were verified with a one-way analysis of variance (ANOVA) using Origin 8. A *p*-value of <0.05 was considered statistically significant. All the experiments were performed in triplicate.

## 3 Results and discussion

### 3.1 Synthesis of QPABA

**Scheme 2:**
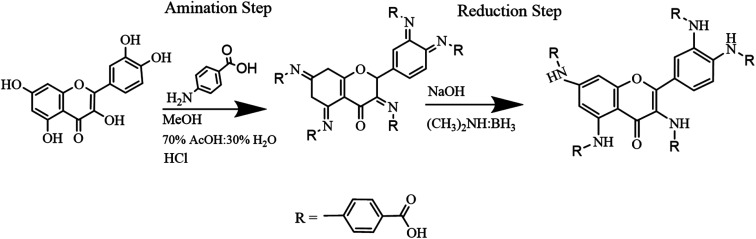
Synthesis pathway of quercetin *para*-aminobenzoic acid (QPABA).

The synthesis of QPABA occurs *via* reductive amination. The initial step of the synthesis is the formation of the imine intermediate (Schiff base) *via* amination (Scheme 2). Water is formed as the side product in the initial step; since water is a solvent in the reaction, it stays in the system and is removed at the end of the reaction. Before the addition of the dimethylamine borane, TLC analysis was performed to identify the formation of the imine compound. The final step is the reductive step that forms the desired product. The TLC is used to monitor the formation of the final product. The side product formed, such as water, is removed during purification. The yield of the synthesis is 95% product with high purity. This is the first time we are reporting the synthesis of QPAB.

### 3.2 Effect of functional groups on QPABA structure

The formation of intramolecular hydrogen bonds in the synthesized QPABA reveals its unique molecular structural properties. The chemical functional groups on QPABA capable of forming hydrogen bonds between a donor and acceptor atom render QPABA soluble and polar, thereby portraying its ability to form specific interactions with transition metals. In the QPABA structure, the intra-hydrogen bonding formed assumes a temporary six-member ring system, which is highly polar (Scheme S1†). The intra-H-bonding and terminal carboxylic groups highlighted in Scheme S1† give QPABA its polar effect and solubility in water.

### 3.3 ^1^H characterization of QPABA

The quercetin derivative, QPABA was synthesized using anhydrous quercetin and *para*-aminobenzoic acid as the starting materials in the methanol, water, and acetic acid solvent system. Initially, the quercetin was dissolved in 200 ml methanol while the *para*-aminobenzoic acid was dissolved in 70% : 30% acetic acid and water, respectively. The quercetin mixture reacted with the *para*-aminobenzoic acid mixture at a stirring rate of 250 rpm for 24 hours. The quercetin *para*-aminobenzoic acid structure was confirmed using ^1^H and ^13^C-NMR.

The ^1^H NMR spectrum (Fig. S1†) depicts three sets of proton signals, including aromatic proton and amide protons of the analyte and a solvent proton signal (Table 1). The formation replaces the hydroxyl groups of an amide bond between the quercetin and *para*-aminobenzoic acid. There are five amide bonds formed in the desired products. The amide protons at ‘3’ and ‘4’ of the B ring of quercetin are in the same chemical environment depicting a triplet at 10.48 ppm chemical shift. The protons are downfield by the presence of the benzene ring. The amide protons from C5 and C3 are depicted at 12.27 ppm and 10.74 are downfield by the carbonyl group on the C ring of the quercetin. Meanwhile, the other amide proton at C7 is showing an 8.36 ppm chemical shift, which is less downfield. The presence of aromatic protons is seen between 6–8 ppm. Finally, the carboxylic acid protons were depicted at 12.71 ppm due to the downfield experience from the immediate presence of the carbonyl. The chemical shift of the amide proton at position 5 on the flavone is a result of intramolecular hydrogen bonding to the carbonyl oxygen atom. A 6-member ring is formed due to intramolecular hydrogen bonding. This tends to alter the proton's chemical environment, causing it to move downfield. The DMSO solvent peak appears as a strong singlet at 2.5 ppm.

**Table 1:** Summary of the ^1^H-NMR chemical shifts of QPABA

Chemical shift (ppm)	Functional group
2.50	DMSO
6.5–8.5	Aromatic protons
10.50	Amine protons (–NH)
11–13	Carboxyl proton (–COOH)

### 3.4 ^13^C-NMR of QPABA

The ^13^C NMR of QPABA showed six-carbon signals within the range of 90–190 ppm (Table 2). The *para*-aminobenzoic acid attached to the parent quercetin molecule at the hydroxyl (OH) positions showed the formation of a new C–N bond. The resultant C–N bond showed a peak at approximately 112 ppm. The benzene ring signals from the *para*-aminobenzoic acid were positioned at 116 ppm and 131 ppm, respectively. Finally, the peak at 170 ppm is assigned to the carbonyl carbon of the carboxylic acid. About the quercetin molecule, the carbonyl carbon from the heterocyclic ring was situated at 180 ppm; this signal confirms the signal reported in the literature. The benzene ring of the C-ring of quercetin had signals from 112 ppm to 120 ppm. The A ring of the parent quercetin molecule showed peaks within the range of 105 ppm to 150 ppm. It was observed that the presence of electronegative atoms such as oxygen and nitrogen shifted the nearest neighbor carbon to a downfield region of the spectrum. The peaks within 10 ppm to 50 ppm are attributed to the solvent peaks. Table 2 provides a summary of ^13^C-NMR peaks. Therefore, it was concluded that the synthesized structure was QPABA as shown in Fig. S2.†

**Table 2:** Summary of ^13^C-NMR chemical shifts of QPABA^28^

Chemical shift (ppm)	Functional group
20–50	Solvent peak
155	Carboxyl group (–COOH)
110–140	Aromatic ring
175	Carbonyl group (C=O)

^13^C NMR (600 MHz, DMSO-d_6_): 176.1, 173.8, 168.9, 166.7, 161, 156.8, 154.1, 152.9, 151.7, 149.0, 147.0, 146.1, 136.0, 135.6, 131.5, 130.3, 122.2, 120.2, 119.4, 118.3, 116.5, 115.8, 112.9, 102.6, 99.2, 94.0 49.0, 44.0, 40.3, 40.1, 31.1, 23.2, 22.8.

### 3.5 Thin layer chromatography (TLC) analysis of the QPABA

The role of TLC analysis was to determine the separation and polarity of the as-synthesized QPABA and quercetin. The TLC silica gel 60 F254 served as the stationary phase and the methanol and ethyl acetate mixture served as the mobile phase. The retention factor (*R*_f_) values calculated were 0.628 and 0.714 for QPABA and quercetin respectively. The *R*_f_ value of QPABA shows pronounced retardation compared to the quercetin. Since silica is polar, the flavonoid derivative with a higher polarity interacts more strongly with the silica and this leads to a shorter distance traveled along the stationary phase. This implies that a lower *R*_f_ value for the QPABA confirmed its enhanced polarity as compared to quercetin. Our results agree with the *R*_f_ values of flavonoid derivatives reported by Yadavalli *et al.*^29^

### 3.6 FTIR characterization of QPABA

**Fig. 1:**
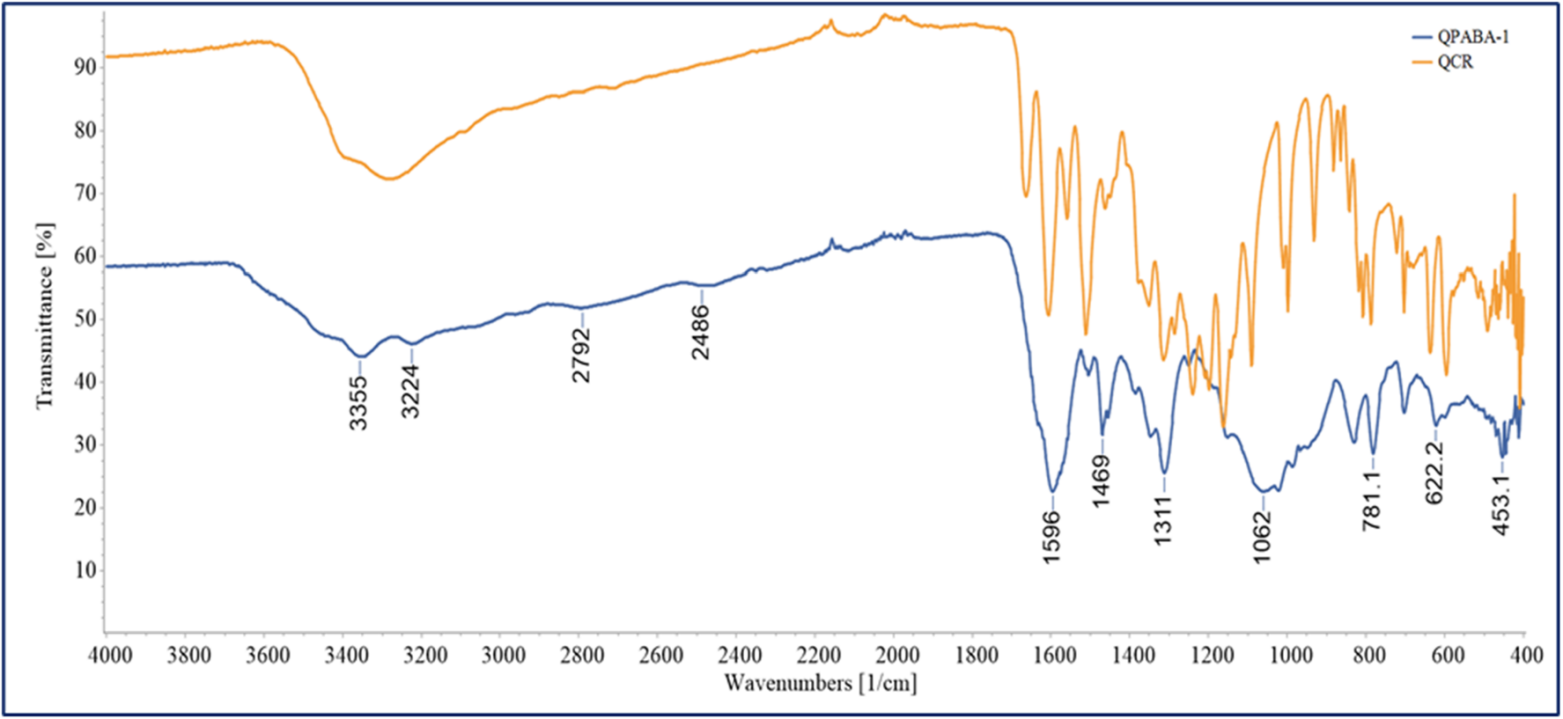
FTIR spectrum of QPABA.

A unique aromatic hydroxyl structure characterizes quercetin. The presence of these hydroxyl and carboxyl functional groups confirms the ability to form transition metal complexes. The OH functional groups on quercetin are transformed into NH groups *via* reductive amination. The vibrational bands of the functional groups are easily identified by FTIR. The FT-IR of QPABA is shown in Fig. 1, indicative of the structural changes of QPABA after synthesis.

The spectra show characteristic peaks of functional groups like OH, CH, C–O, and C=O bands following the reported spectra in the literature. The FTIR spectra of QPABA are illustrated in Fig. 1, and it is evident that the formation of the QPABA structure was confirmed by the presence of a broad peak from 3500–3000 cm^−1^, indicating the existence of –OH stretch, which is intrinsic to *para*-aminobenzoic acid (–COOH), and 1711 cm^−1^ carbonyl group C=O (–COOH stretching vibration), 1658 cm^−1^ carbonyl group C=O (stretching vibration from the C-ring), 1539 cm^−1^ (N–H bending), the peak at 1500 cm^−1^ is a characteristic of the aromatic ring (C–C stretching), strong band identified at 1242 cm^−1^ indicates the existence of C–N bonds. The comparison of quercetin (QCR) and QPABA reveals the differences in the functional group present.^30–32^

### 3.7 Effect of concentration on the formation AuNFs and AuNS

The green synthesis of anisotropic gold nanoflowers and nanostars is sensitive to the reaction parameters such as the concentration of gold precursor, reducing/stabilizing agent, the mixing ratio of starting reactants, and the reaction time. In this concentration-based study, the concentration effect of QPABA was thoroughly investigated on the growth of both AuNFs and AuNS. To understand the growth mechanism of AuNFs, a one-pot technique experiment was performed to achieve AuNFs and AuNS selectively. Five sets of 50 ml vials were used for experiment 1. In each vial, 400 μl of 5 mM gold stock was transferred followed by the addition of QPABA (200, 400, 600, 800, 1000 μl) into each independent vial. Each reaction vial was diluted with 1000 μl of ultrapure water. In the second series of experiments, the concentration of QPABA in each vial was fixed at 400 μl, and the gold stock added varied from 200 to 1000 μl. The resultant reaction was diluted with ultrapure water after the instant color change from yellow to wine.

### 3.8 TEM characterization

**Fig. 2:**
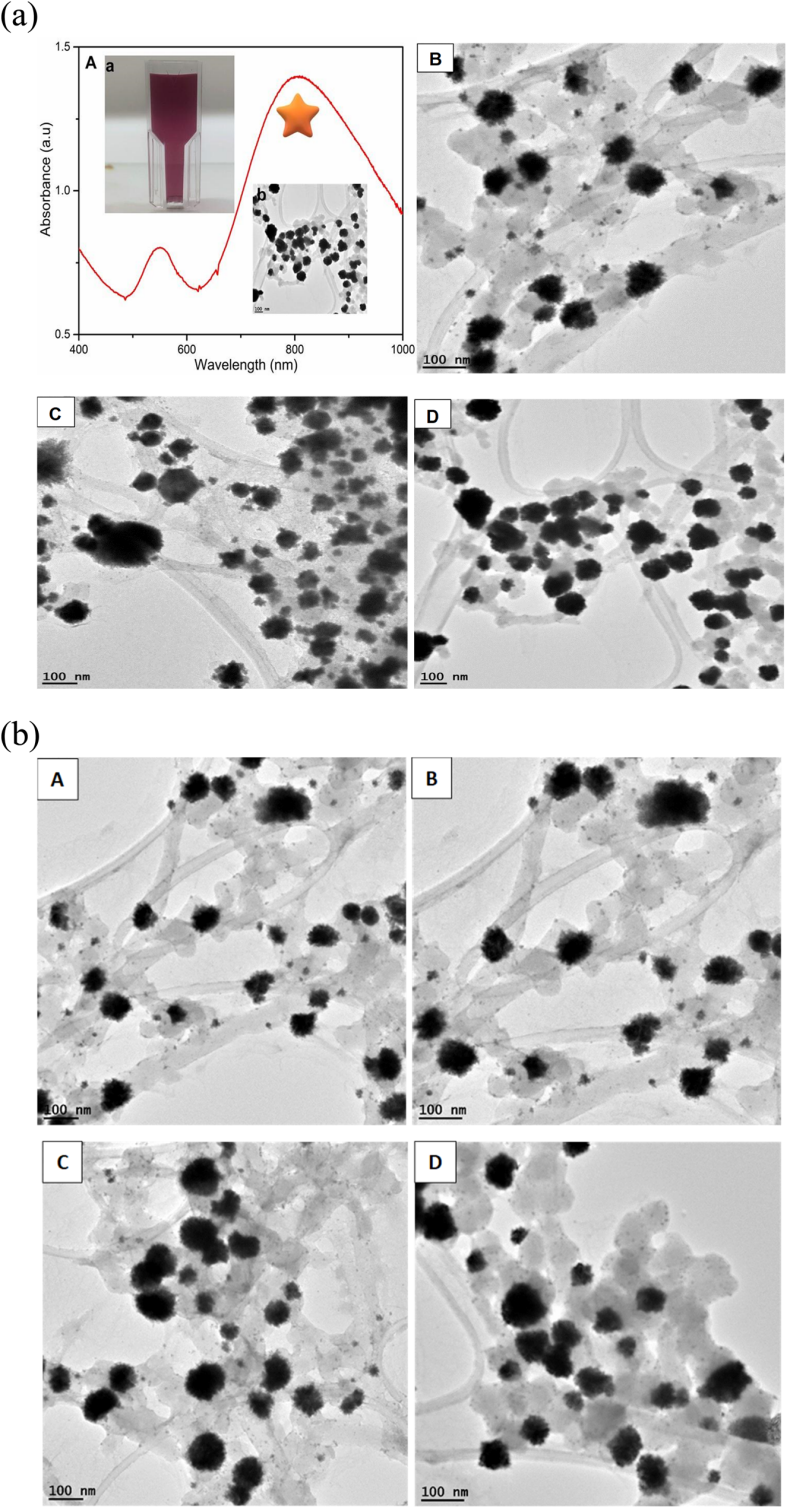
(a) UV-vis spectra of AuNFs showing the absorption of AuNFs (A), inset: digital image of AuNFs (a), TEM images of AuNFs (b) and TEM images of AuNFs at 100 nm (B, C, and D). (b) TEM images of AuNFs (A–D) at a scale bar of 100 nm.

The synthesis of gold nanoflowers (AuNFs) always requires high energy to reduce Au^3+.^ In this work, AuNFs were formulated using the functionalized water-soluble QPABA at room temperature. One-pot synthesis was adopted, where QPABA and HauCl_4_·3H_2_O reacted in a vial without the addition of capping and stabilizing agents. Hence QPABA acted as both the reducing agent and capping agent to produce AuNFs, as shown by the TEM images in Fig. 2a, b and S4.† It is worth mentioning that, as the concentration of QPABA increased, the yield of the AuNFs increased dramatically to nearly 100%, and the morphology of AuNFs in terms of the enhanced branching of the reproducible nanoparticles was demonstrated. The synthesized AuNFs exhibited flower-like branched structures in Fig. 2(a) B, C and D, with near solid infra-red (NIR) absorbance centered around 800 nm as well as a less intense band centered at around 550 nm, as depicted in Fig. 2(a) A.

### 3.9 X-ray diffraction characterization (XRD) and energy-dispersive X-ray spectroscopy (EDS) characterization

**Fig. 3:**
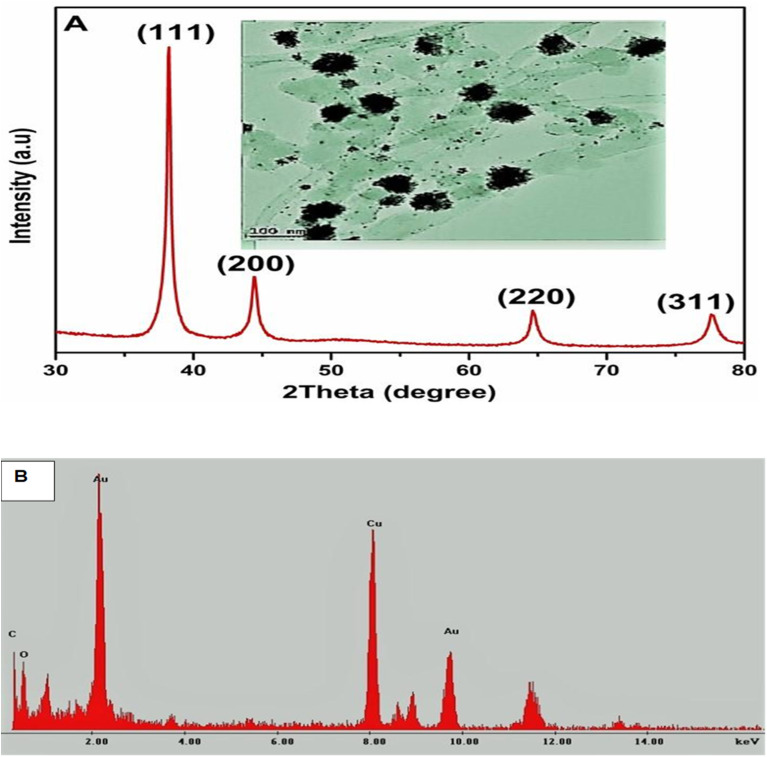
(A) XRD of AuNFs and HRTEM images of AuNFs (inset), XRD image shows the crystalline structure of tested and green synthesized gold nanoflowers. (B) EDS Analysis of AuNFs.

An X-ray diffractometer was employed to probe the crystalline nature of the gold-synthesized nanoflowers and nanostars. The XRD patterns in Fig. 3 display sharp peaks at the 2*θ* position with lattices (111), (200), (220) and (311). The lattices from the XRD are characteristic of the face-centered cubic structure of the AuNFs. The lattices were deduced from the following Braggs angle at 2*θ* position from 28°, 38°, 44°, 64°, and 77°, respectively, for the AuNFs sample at room temperature. However, the additional peak at 28° may be due to phytochemical capped onto the synthesized AuNFs and AuNs.

The XRD patterns of the AuNFs match the database of JCPDS file no. 04-0784, confirming that the synthesized AuNFs are pure crystalline. The Debye–Scherrer's equation was utilized to compute the crystalline domain size of the AuNFs to determine the FWHM of the (111) ‘Bragg's reflection from the X-ray diffractogram.^33^ The synthesized AuNFs were found to have average crystallite sizes between 20 to 50 nm. The particle size from the XRD results is in close agreement with the average sizes indicated by the TEM images. This confirms and connotes the flowerlike formation of gold nanoparticles.^34^ The EDS was used to characterize the elemental mapping of the synthesized gold nanoflowers and the gold nanostars. The analysis of this technique indicated the elemental pattern of the gold nanoflower specimen. The EDS profile of the as-prepared gold nanoflower (Fig. 3B) clearly shows that the composite of the AuNFs consisted of Au, C, O, and Cu. The C and Cu peaks are attributed to the copper–carbon grid. The C–Au's elemental mappings depict that the copper and gold composite confirm that copper and gold are the two homogenous elements established at the crystallite level from the above measurements.

### 3.10 Optical properties of AuNFs and AuNS

**Fig. 4:**
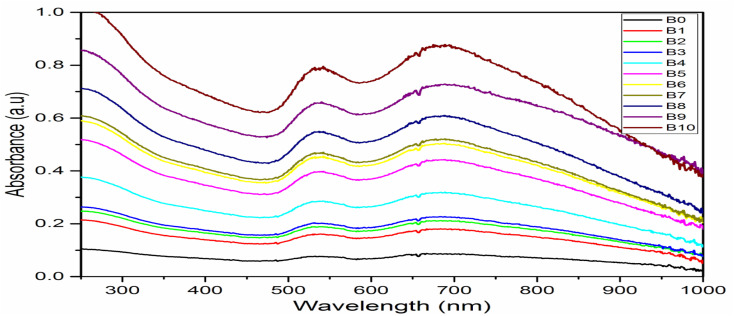
UV-vis spectra of AuNFs showing SPR peaks at 548 nm and 700 nm attained by monitoring reaction times corresponding to gold nanoflower production.

**Fig. 5:**
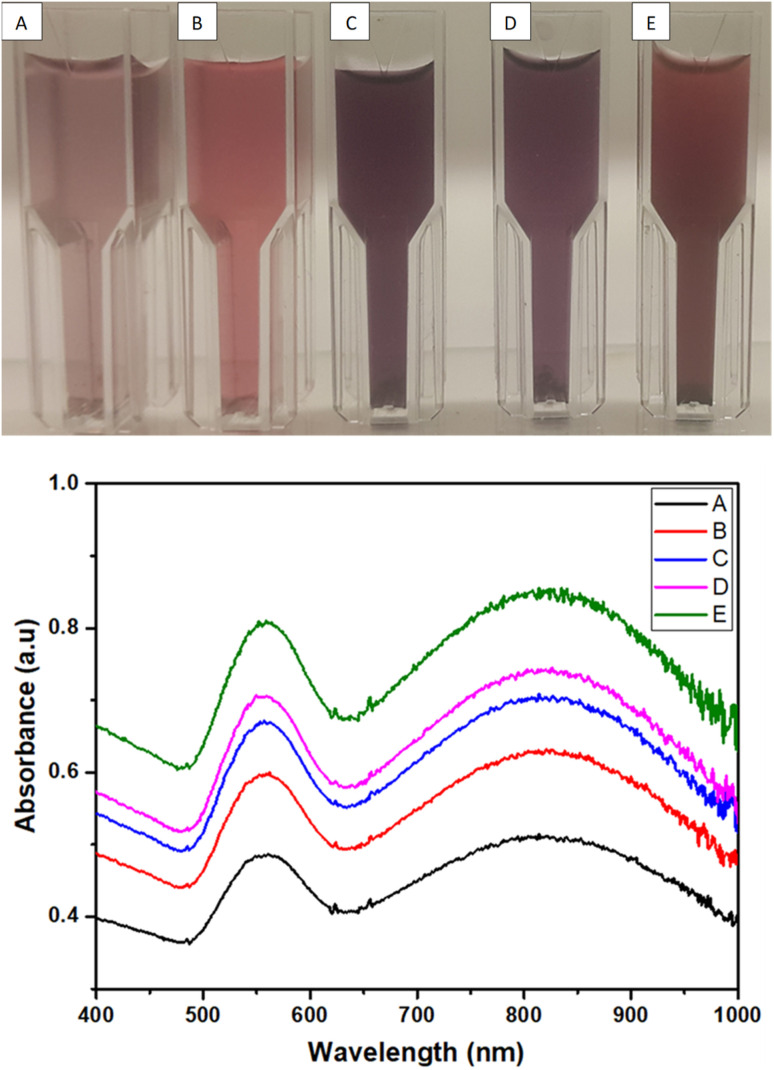
Digital images of AuNs (A, B, C, D and E) and the corresponding UV-vis spectrum for the formation of AuNs with SPR peak at 558 nm and 825 nm.

The formation of AuNFs and AuNSs was confirmed using UV-vis spectroscopy (Fig. 4 and 5 respectively). From the measurement, the SPR peak of nanoflowers was 50 nm in diameter and was located at 550 nm. The smaller gold nanoflowers experienced a shifted SPR peak towards the shorter wavelengths of the spectrum. Nonetheless, the SPR peak of the irregular-shaped nanoflower was indicated at 520. It was demonstrated that nearly AuNFs particles of an average diameter of 70 nm were absorbed in 550 nm. Understanding the SPR peaks concerning the average size of the gold nanoflower particle lies in the domain of the Plasmon hybridization theory. Morphologically, nanoflowers are described as arm-like and branched particles.^35,36^ During the growth process, it was observed that the rate of reduction decreased because of the depletion of the limiting reactant (the Au precursor salt) over time. The progress of the reaction and the formation of gold nanoflowers between 50 nm to 80 nm were monitored using the time-based UV-vis spectra measurement. It has been reported that there are three unique growth phases of nanoflowers which includes: the reduction of Au^3+^ to Au^0^ nanocrystals, followed by the agglomeration of the primary Au nanocrystals into intermediate agglomerates, and the anisotropic growth of the agglomerates into flowerlike nanostructures.^37^ The growth process allows the gold nanoflowers to extend their arms toward more energetically favorable directions, resulting in the anisotropic gold nanoflowers agglomerate. This conclusion resulted from the final growth process. With further supporting evidence, the TEM images revealed the anisotropic growth of the gold nanoflower from the synthesis at 0.5 min to 60 min (Table S1†) into highly branched Au nanostructures. The growth of flowerlike Au nanostructures from 0.5 to 60 min (from diameter 80 nm at 60 min to 50 nm at 20 min a) agrees with the red shifting of the SPR in the visible light region. The reaction was completed in 60 min. This is consistent with results reported in literature.^24,38^ The kinetics of the formation of the flower-shaped morphology using UV-vis absorption spectra and the optical properties of the nanoflowers are presented in this section. During the growth process, the aqueous HauCl_4_ solution was pale yellow before the addition of the bi-functional QPABA. There was an instant color change upon the addition of the QPABA. As time progressed, the color of the reaction occurring in a one-pot setting changed from slightly yellow to reddish color.

According to the changes in the UV-vis absorption spectra, the samples at different stages were purified and further characterized using HRTEM. Fig. 2b shows the HRTEM image of initially shaped gold nanoflowers taken at 5 min. The core grew into quasi-spherical with a diameter of nearly 50 nm, while their tips appeared small and short. UV-vis spectra showed one absorption peak at 550 nm resulting from the SPR of the core. The increase of time led to the significant growth of petals from the core of the gold nanoflower. Meanwhile, the UV-vis absorption spectrum revealed two absorption peaks: a weak one at 548 nm and a strong one at 700 nm, which corresponded to the SPR modes of the cores and the tips, respectively. The tip SPR band showed a red shift during the reaction. During the quenching of the reaction at 60 min, the growth of the gold nanoflowers with an average size of about 80 nm was uniform, and their tips were petal-like in structure (Fig. 8). The reaction proceeded beyond the 60 min mark (Table 3), and no significant changes in shape and size of the gold nanoflowers were observed.^24,39^

**Table 3:** Comparative analysis of flavonol and the effect of ligand (PABA) incorporation on the anisotropic property of gold nanoparticles

Flavonol	Gold-NP synthetic method	Physiochemical properties (size and shape)	Ref.
Myricetin	Ultra-sound	Spherical shape, size > 50 nm	Mohan *et al.*^44^
Fisetin	Nano-precipitation	Spherical shape, size 140–200 nm	Sechi *et al.*^45^
Kaempferol	Chemical reduction	Spherical shape, size 16.5 ± 2.5 nm	Raghavan *et al.*^46^
Quercetin	Green chemical reduction	Spherical shape, size 38 nm	Divendiran *et al.*^47^
QPABA	Chemical reduction	Nanoflowers and nanostars shape, sizes 20–50 nm	This study

### 3.11 TEM and size morphology characterization of AuNs and AuNFs

**Fig. 6:**
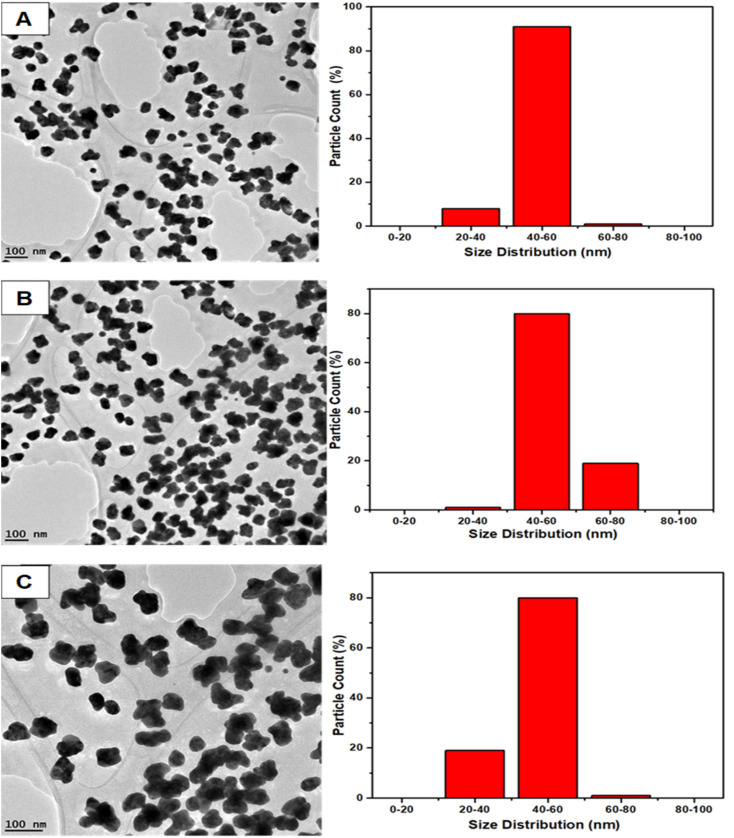
Size distribution of green synthesized gold nanostars (A–C) showed a narrow range. Highly reproducible under ambient conditions. Mainly identified nanostars with 20–80 nm diameter.

**Fig. 7:**
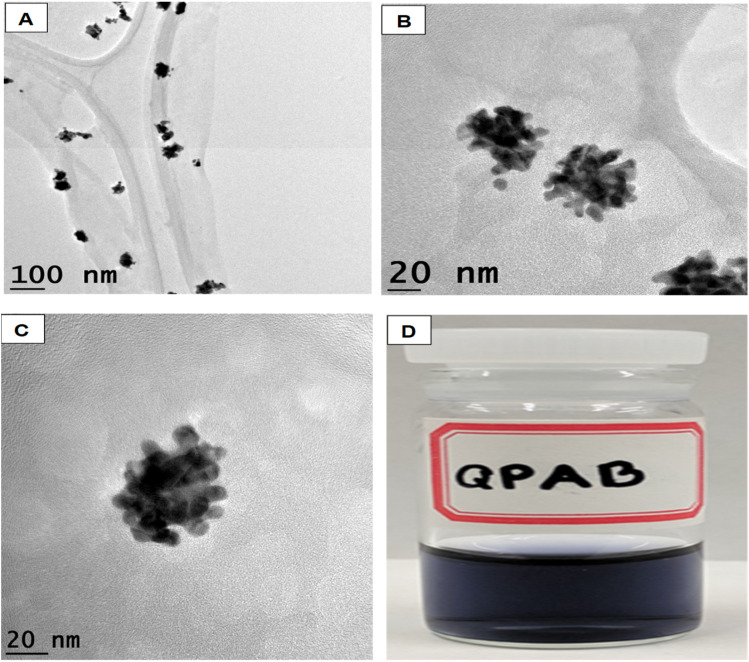
TEM image of AuNSs (A). HRTEM image of AuNFs (B and C). Digital image of AuNSs (D).

The representative TEM images and corresponding size distribution histograms of AuNs are shown in Fig. 6. The AuNFs TEM images were formed using 5 mM HauCl_4_·3H_2_O solution and 5 mg QPAB solution. Plate-like images with a size range of 50–80 nm. Fig. 6A–C exhibited the formation of gold nanostars. The formation of AuNs with long extending pointed arms as a key characteristic of gold nanostars was exhibited in Fig. 7A–C. Further reduction of the precursor concentration to 2.5 mM produced AuNFs (Fig. 7D) with a branched morphology and average size within the range of 40–60 nm. These nanostructures have a dense core and highly branched petals. It has been reported by several studies that AuNFs with different branching are driven *via* a trend where the concentration of the gold decreases and the size of the AuNFs increases in the presence of the QPAB ligand. The core of the gold seed with its sharp branches is a major contributing factor to the high surface-to-volume ratio indicating their potential biological and therapeutic applications. In this regard, Au nanoparticles serve as a scaffold for biological applications due to their good biocompatibility and low cytotoxicity. Furthermore, gold nanoflowers are useful in Raman spectroscopy for probing living cells' functionalities. Previous studies have shown that Au nanoflowers (AuNSs) with sizes around 60–80 nm have the highest efficiency for SERS using red (633 nm) or near-infrared (785 nm) excitation.^40,41^

**Fig. 8:**
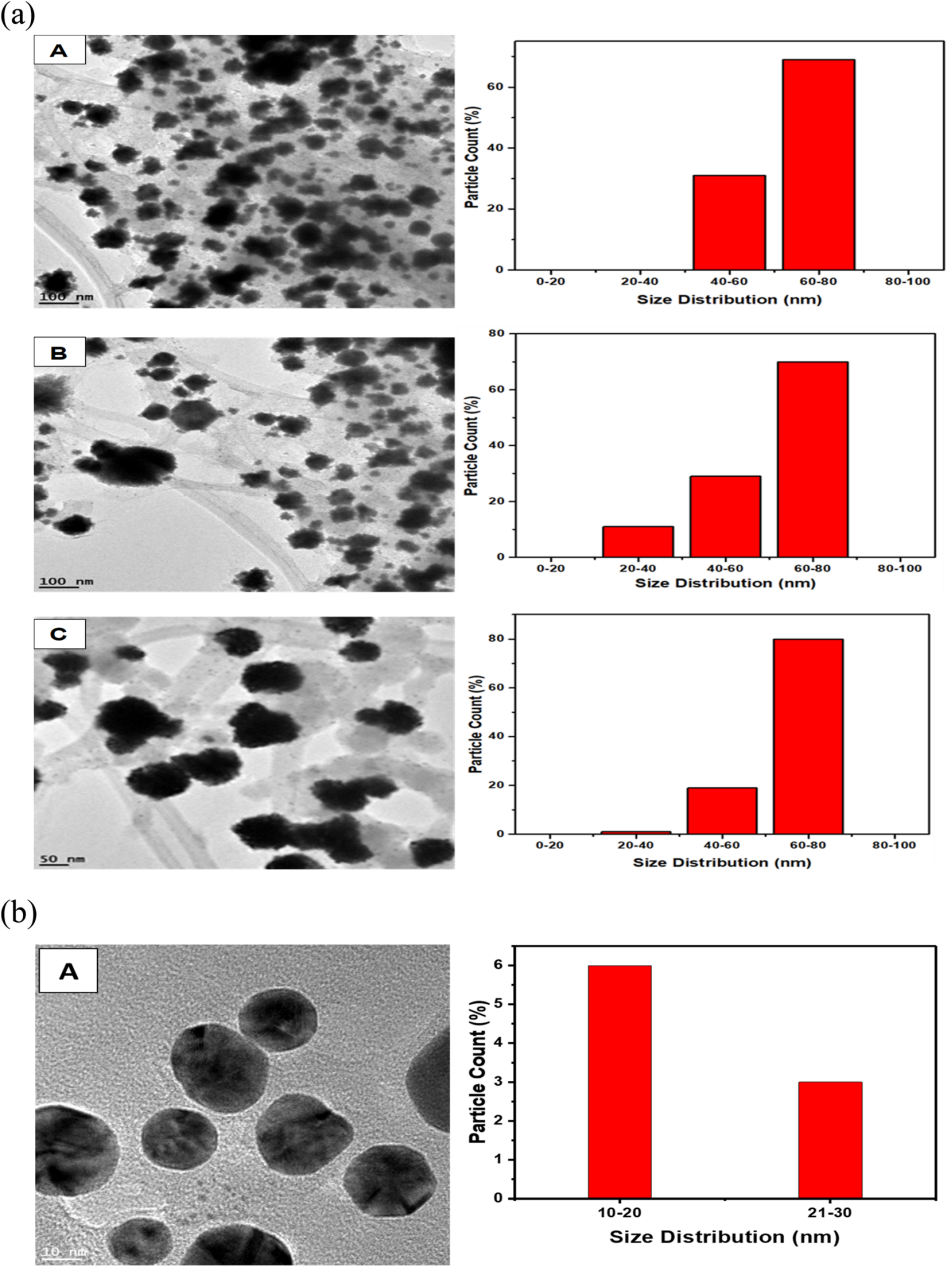
(a) TEM image of AuNFs with the corresponding size distribution bar graph of particles in the image with the size distribution of 50–80 nm in diameter (A, B, and C). (b) TEM image of spherical AuNPs with the corresponding size distribution graph and average size of 18.4 nm in diameter.

The growth of gold particle size was controlled *via* the concentration of a capping agent. In this study, QPABA acted as both capping and reducing agents for directing the sizes of resultant gold nanoflowers. The molar concentration of QPABA had significant effects on the size and morphology of the gold nanoflowers. Fig. 4 shows a shift in the absorption spectra of gold nanoflowers synthesized at different concentrations and reaction times. It was observed that an increase in the concentration of QPABA corresponded to a shift in the absorption peak towards a lower wavelength, indicating a decreased particle size. Furthermore, the shift in the absorption peak was attributed to the localization of surface plasmon resonance of the gold nanoflower. The synthesized gold nanoflowers exhibit a narrow size range between 20 to 80 nm (Fig. 8A) due to the ability to control the concentration of QPABA. The optical studies of gold nanoflower revealed that the shift in absorption peak suggests that increasing the concentration of QPABA leads to an increase in the energy bandgap due to decreasing the particle size.^40,42,43^ However, using the same the synthetic method of gold nanoparticles by reacting quercetin with gold(iii) chloride; AuNFs was not formed but instead spherical gold nanoparticles with an average size of 18.4 nm was produced as depicted in Fig. 8B and S3†. Hence the modification of quercetin with *para* amino benzoic acid played a significant in the formation of gold nanostars and nanoflowers.

### 3.12 Possible mechanism for the formation of AuNFs and AuNS

**Scheme 3:**
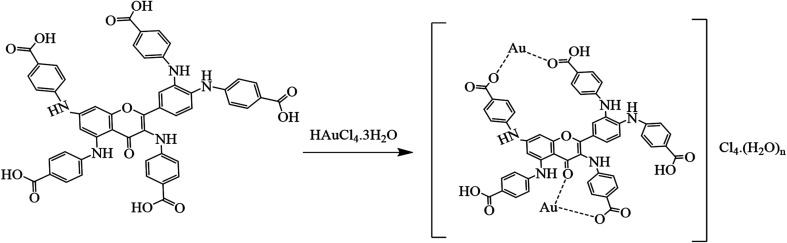
Probable mechanism of gold nanoflowers and nanostars formation *via* the chemical reduction in the presence of QPABA.

The mechanism underlying the formation of nanoflowers and nanostars is concentration-driven between QPAB and the gold metal precursor. The carboxylic group of QPABA enables it to form a complex with the gold precursor *via* chemical reduction (Scheme 3). It has been reported that the source of antioxidants in quercetin is attributed to the presence of the hydroxyl group. In this regard, the terminal carboxylic groups are the driving force of metal complexation, leading to different shapes and sizes of gold nanoparticles. In this work, the gold precursor was kept constant, the QPABA ligand was varied, and the growth of AuNFs was observed through growth phases. This led to a complete reduction of the gold metal precursor. This mechanism can qualitatively be observed during the initial growth stage, where Au^3+^ are reduced to Au^0^ nanoflowers, and it then proceeds furthermore into an agglomeration of the primary particles to form intermediate agglomerates. We have demonstrated that increasing the concentration of QPABA in the presence of gold, ions results in complete chemical reduction.^38,40^

Shape-directing Ligands play a significant role in the growth mechanism of nanoparticles. Table 3 illustrates the physiochemical properties of using the different flavonols. The modification of quercetin using *para*-aminobenzoic acid as a ligand led to the gold nanoflowers and nanostars and the overall solubility of the QPAB. Some reports have shown the impact of incorporating the surfactants/ligands in nanoparticle synthesis which controls the shape and properties of the resultant particles.^18,19^ In all, gold nanoflowers with outstanding properties have been achieved due to the modification of quercetin with *para*-aminobenzoic acid.

### 3.13 Antifungal activity of AuNFs and spherical AuNP against *Fusarium solani* spp

**Fig. 9:**
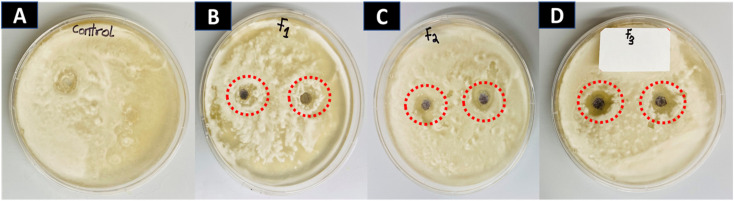
Effects of AuNFs and AuNPs on mycelial growth of *Fusarium solani* spp. Images represent the five days after treatment of AuNFs against *Fusarium solani* spp. (A) Control (B); 100 μg mL^−1^ (C); 150 μg mL^−1^ (D); 200 μg mL^−1^.

To control the activity of soil-borne *F. solani*, the mycelia growth of the fungi was treated with AuNFs in a dose-dependent manner and assessed daily in comparison to the control. Fig. 9 illustrates the inhibitory effect of AuNFs on the mycelial growth of *Fusarium solani.* The antifungal activities of AuNFs were quantified and statistically analyzed using the zone of inhibition.

Table 4 shows the zone of inhibition of *Fusarium solani* based on the dose treatment of AuNFs. The AuNFs significantly inhibited the growth of *Fusarium solani* as compared to the control (*p* <0.05). Among the AuNFs concentrations tested, 200 μg mL^−1^ showed the highest inhibition at 11.00 mm ± 0.15 against *Fusarium* spp. In addition, the least effective dose of AuNFs was 100 μg mL^−1^ which corresponds to a 4.00 mm ± 0.09 zone of inhibition against the growth of *Fusarium solani*. The MIC values of the AuNFs were estimated statistically to be 125.7 ± 0.22 μg mL^−1^ against *Fusarium solani*. According to our findings, the AuNFs are more effective than spherical AuNPs against the growth of the plant pathogen *Fusarium solani*. The treatment of spherical AuNP against *Fusarium solani* did not show any zone of inhibition as displayed in Fig. S6.†

**Table 4:** Zone of inhibition of *Fusarium solani* at different dose treatments of AuNFs

Fungus	AuNFs nanoparticle treatment	Zone of inhibition (mm)
*Fusarium solani* spp	100 μg mL^−1^	4.00 ± 0.09
	150 μg mL^−1^	7.00 ± 0.25
	200 μg mL^−1^	11.00 ± 0.15

The efficacy of AuNFs as antifungal agents is attributed to their small size-to-volume ratio. Several reports have demonstrated the influence of shape as a contributing factor to an antimicrobial property. Among effective nanomaterials, gold branched nanomaterial was fabricated owing to its.^3,18^ Biocompatibility, less toxicity, and highly stable with several applications in antimicrobial treatment.^3^ The antifungal mechanism of AuNFs was due to the small particle size to volume ratio, oxidative stress induction, and the release of the metal ion. Oxidative stress is usually caused by radicals including superoxide (O^2−^) and hydroxyl radicals (–OH).^18,19^ In this study, the metal ions in the solution together with the flowerlike shape of the particles were responsible for the death of mycelial growth. The presence of an inhibition zone on the media indicates the antifungal activity of AuNFs. The extent of inhibition was tied to the concentration of AuNFs. AuNFs possess promising biocidal activity against *F. solani*. Due to environmental concerns about synthetic fungicides, bio-control agents are promoted for plant disease management. Several treatments have been reported as promising biological control agents against some pathogenic fungi such as *F. solani*.^3^ These results propose the use of AuNFs as an effective green antifungal activity for inhibiting diseases caused by *F. solani*.

## 4 Conclusion

We have demonstrated a high-yielding one-pot synthesis of gold nanoflowers and nanostars at room temperature using QPAB as the bi-functional reducing and stabilizing agent. The QPABA was synthesized using a two-step greener reductive amination approach. The final product, QPABA, was characterized successfully and used to synthesize anisotropic gold nanoflowers and nanostars. The morphology of gold nanoflowers and gold nanostars can be modulated by adjusting the molar ratio of QPAB to the gold metal precursor. The average size of the Au nanoflowers was tunable by controlling the concentration of the gold precursor and the QPAB ligand. After completing all growth phases, the gold flowerlike branching nanostructure shows an increase in the surface area to volume. Both treatments of AuNFs and AuNP against *Fusarium solani* demonstrate that AuNFs is an efficient antifungal activity against *F. solani*. Thus, the findings in this work are useful for several applications in plant disease management. The difference in shapes and size connotes different functions for useful applications such as drug delivery with improved solubility.

## Conflicts of interest

There are no conflicts to declare.

## Supplementary Material

RA-012-D2RA05478G-s001
