# Supplementary material for: Ligand effect on controlling the synthesis of branched gold nanomaterials against fusarium wilt diseases
Source: RSC Adv. 2022 Nov 7;12(49):31855–68. doi: 10.1039/d2ra05478g (PMC9639171; doi:10.1039/d2ra05478g)
Supplement: RA-012-D2RA05478G-s001 [file RA-012-D2RA05478G-s001.pdf]

Electronic Supplementary Material (ESI) for RSC Advances.
This journal is © The Royal Society of Chemistry 2022

Supporting Information

Ligand Effect on Controlling the Synthesis of Branched Gold Nanomaterials Against Fusarium Wilt Diseases

Francis J. Osonga, Gaddi B. Eshun & Omowunmi A. Sadik *

BioSensor Materials for Advanced Research and Technology (The BioSMART Center),

Chemistry and Environmental Science Department, New Jersey Institute of Technology,

University Heights, 161 Warren Street, Newark, NJ 07102

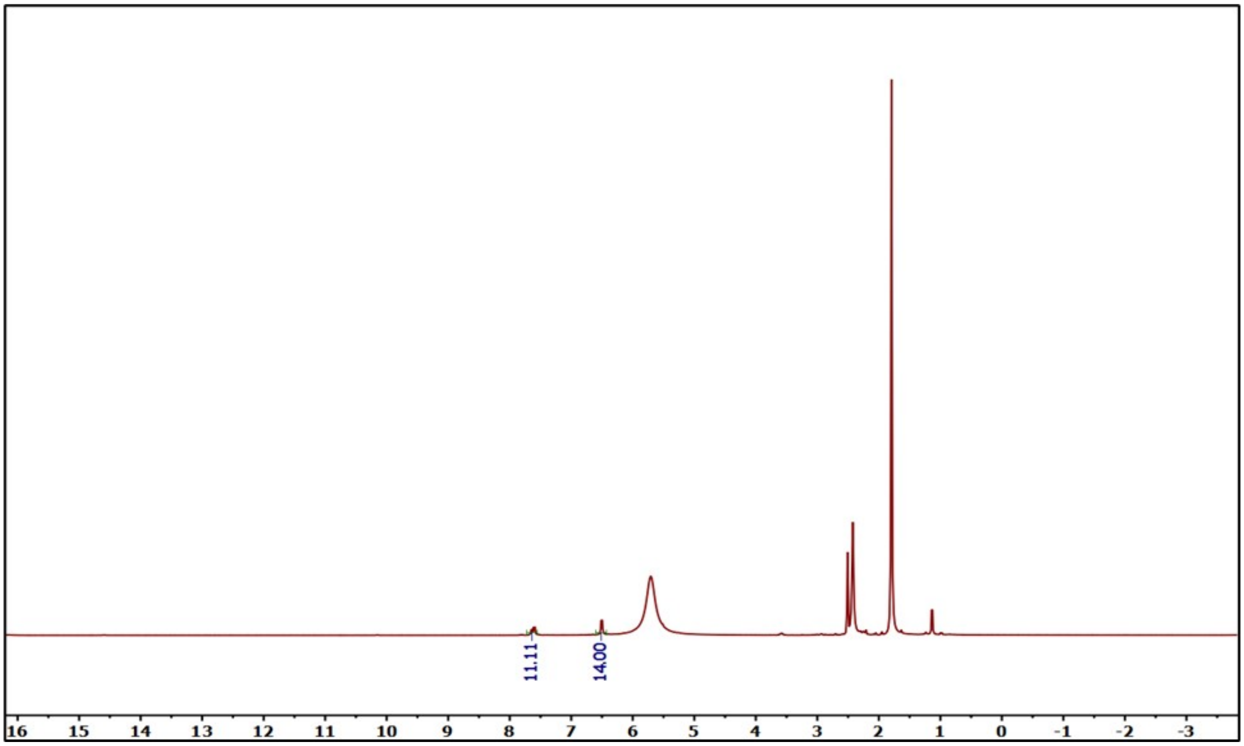

Figure S1: ^{1}H-NMR spectrum of Quercetin-Para aminobenzoic acid

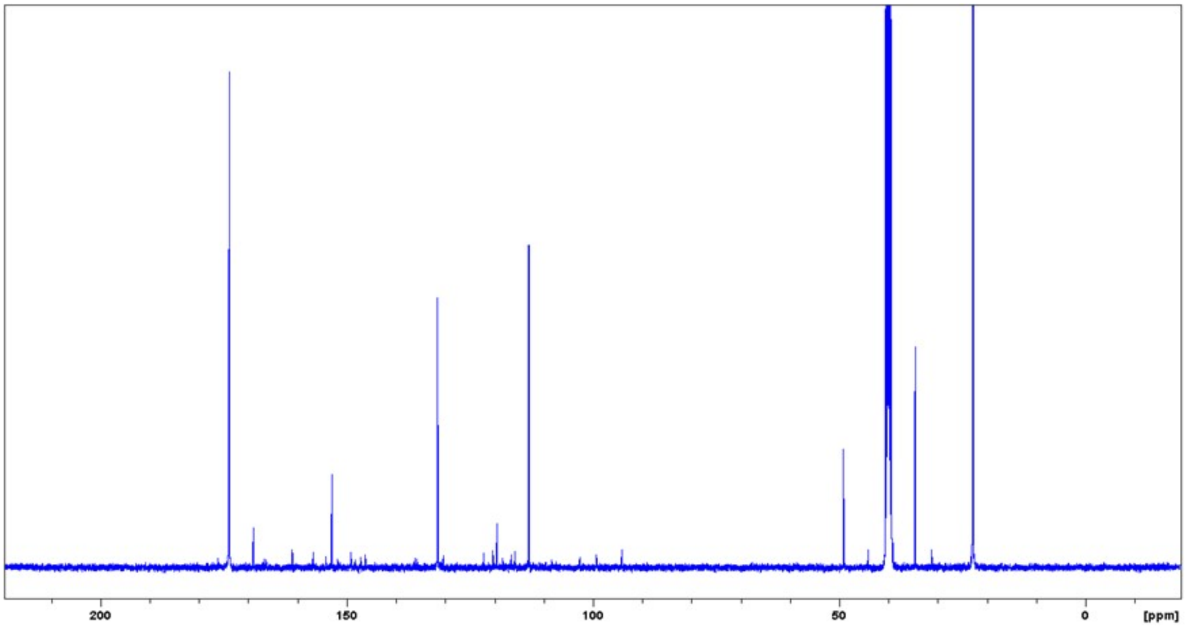

Figure S2: [13] C-NMR Spectrum of Quercetin Para aminobenzoic acid. (QPABA)

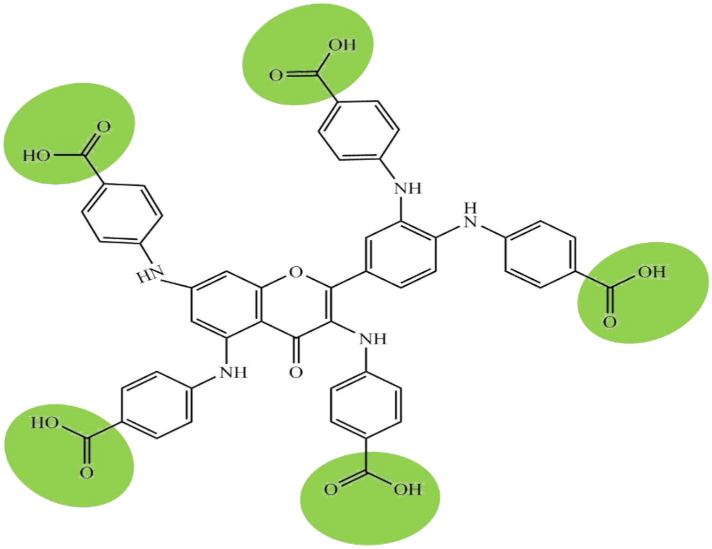

Scheme: S1 Schematic representation of QPABA and showing -NH and -COOH groups for intramolecular hydrogen bonding

Table S1: Time-varying experiment shows 11 samples between 30 sec to 1hr reaction time. Constant concentrations throughout using a 4:1 ratio of reagents

Sample	Reaction time (min)	QPABA (µl)	$HAuCl_4$ (µl)
B0	0.5	1000	250
B1	5	1000	250
B2	10	1000	250
B3	15	1000	250
B4	20	1000	250
B5	25	1000	250
B6	30	1000	250
B7	35	1000	250
B8	40	1000	250
B9	45	1000	250
B10	60	1000	250

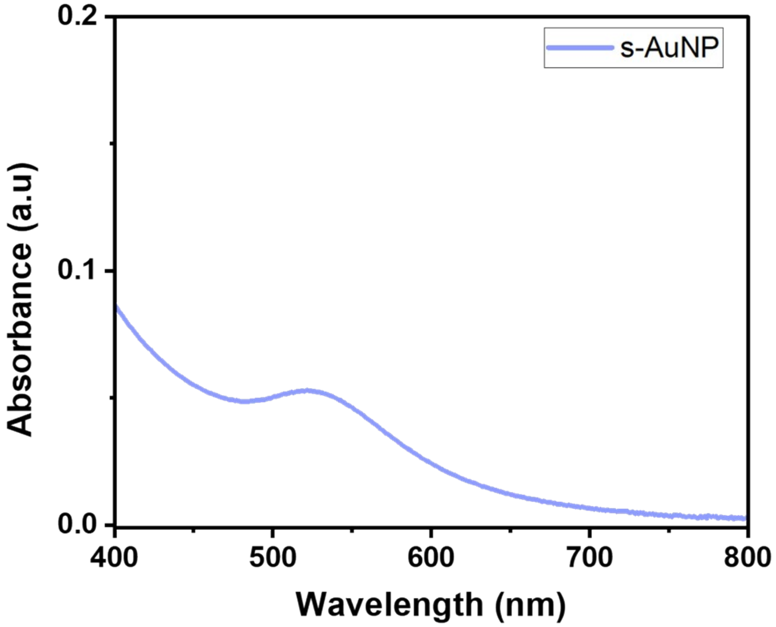

Figure S3: UV-Vis of spherical gold nanoparticles using quercetin.

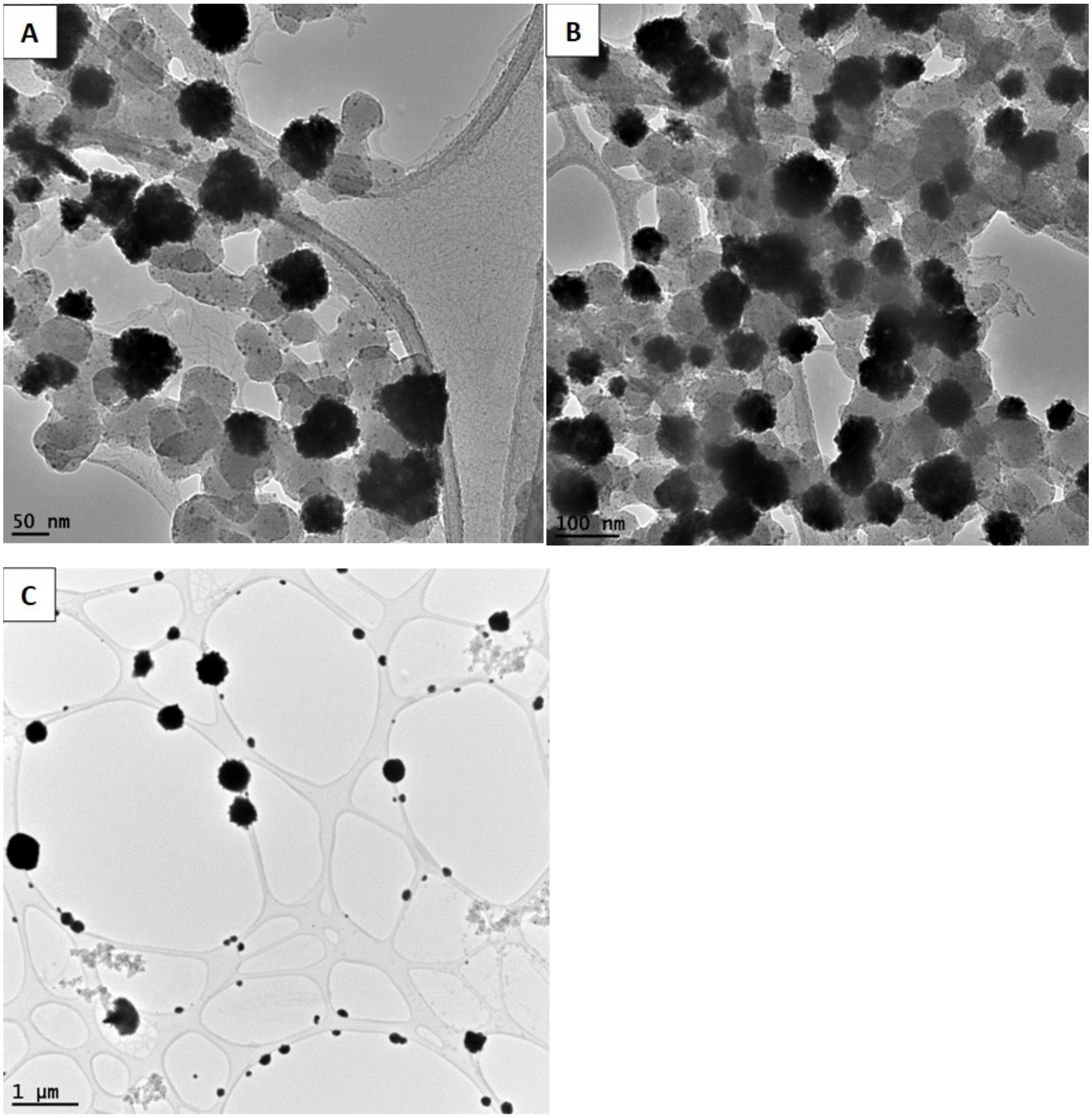

Figure S4: TEM images of AuNFs at 50nm (A) ,100 nm (B) and 1 µm (C).

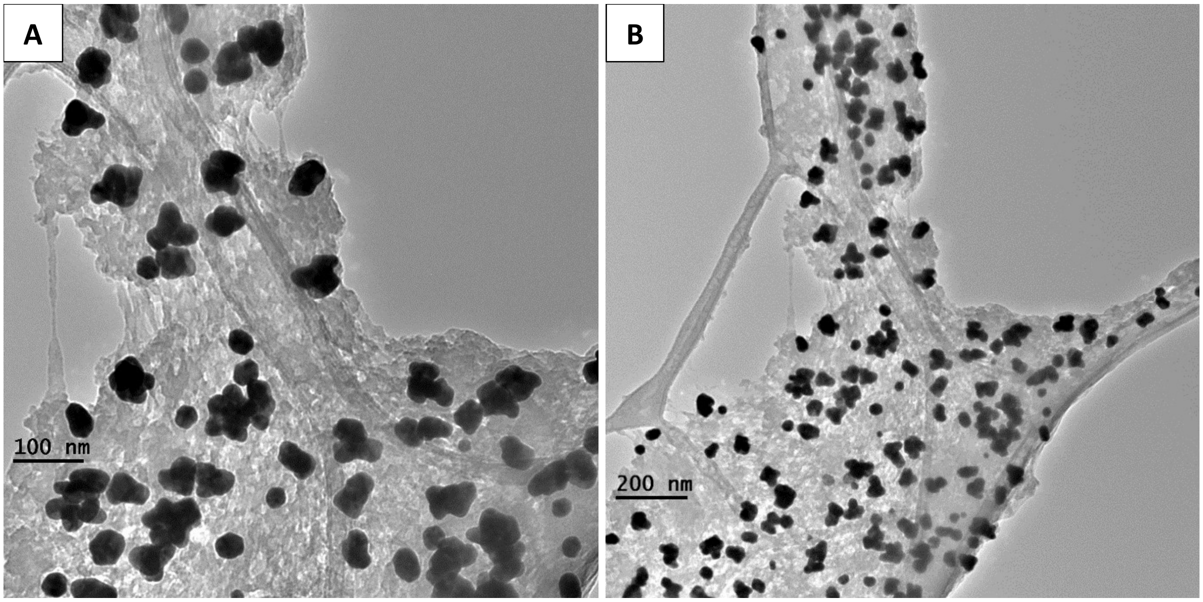

Figure S5: TEM images of AuNS at 100nm (A) and 200 nm (B).

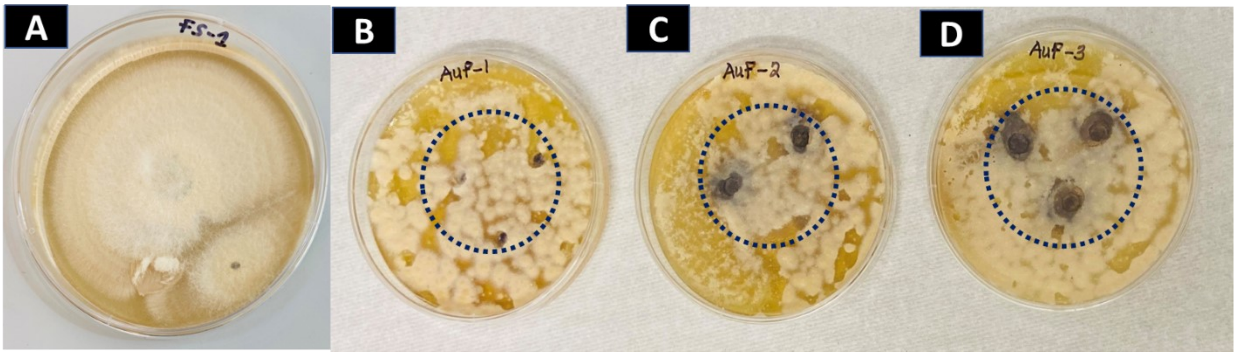

Figure S6: Effects of AuNPs on mycelial growth of fusarium Solani ssp . Images represent the five days after treatment of AuNFs against Fusarium solani spp. (a) control (b); 100 µg/mL (c); 150 µg/mL (d); 200 µg/mL
